# Rose colored glass

**DOI:** 10.1017/S1478951526102181

**Published:** 2026-04-07

**Authors:** Lauren M. Hinkley

**Affiliations:** Johns Hopkins Univeristy School of Medicine, Baltimore, MD, USA

It only registered to me how long I had been standing in the aisle when I realized that my fingers were no longer numb from the walk across the parking lot in the bitter December cold. I was familiar with this aisle: the beverages aisle at Safeway, with different brands and flavors of sparkling water stretching halfway down. I visited frequently, having become a devoted consumer of sparkling water since starting medical school – anything to stay awake. My visits were usually quick and thoughtless, grabbing a pack of whatever brand had a sale on my favorite flavors: cherry, lime, and, when those weren’t available, raspberry. Today, however, the choice felt overwhelming. Somehow, I had never really noticed just how many flavors there were. I cut myself some slack – it was easy to choose a flavor for studying, but much harder to choose one I would forever associate with loss.

***

As a third-year medical student, I am required to take several short courses to supplement my clinical education. Earlier that day, I finished my last session in the course titled “Palliative Care,” where, among other things, we practiced breaking bad news and caring for patients at the end of life. After a particularly difficult session during which we practiced breaking bad news to devastatingly realistic patient-actors, the palliative medicine physician leading my group offered advice on how she copes with the patient deaths. *Have a ritual*, she suggested. *On days when I lose a patient, I go home and toast to them and their life with a glass of wine, or sparkling water if I’m on call.*

So, standing in that aisle of Safeway, I thought about my patients who had died and wondered what flavor sparkling water I should designate to toast to them, what flavor I should forever associate with these losses.

On the one hand, I wanted it to be something I liked – enjoying the toast felt like the only way to truly honor my patients who had passed. On the other hand, I was hesitant to allow any of my favorite flavored beverages become inseparably intertwined with feelings of loss. The thought felt like a betrayal – after they shared their most vulnerable final moments with me, how could I now withhold something as trivial as a beverage flavor? But I forced myself to push through the feeling, aware that I had not been doing a good job of establishing emotional boundaries in my role as a medical student. This was especially true in the weeks following my Internal Medicine clerkship, when I kept returning to the charts of my three sickest patients, continuing to monitor them even after they were discharged on hospice. One by one, their deaths appeared in the medical records, all within two weeks of my clerkship finishing. Their losses devastated me. So, it felt important that I begin establishing these boundaries, even if just by not using my favorite flavor for the toasts.

But what did that leave me with? How could I possibly decide whether vanilla-orange felt more fitting than key lime to use in a toast to the deceased? I couldn’t imagine a situation in which limoncello or berry-tastic *tasted* like loss.

I decided to try a different approach. I could not imagine any of the flavors ever tasting just right, but lucky for me, corporate marketing had long decided that flavors are associated with colors, giving me another sense off which to make the decision. So, the question became: what color *felt* like loss? And this time, the answer was easy.

Pink.

Pink was the color of the bandana still tied around my backpack in honor of my friend, Bernard. When his science fair rocket misfired directly into his chest and killed him before the medical transport helicopter could reach him, his friends decided that we would wear pink bandanas in memory of him. Mine has lived on the handle of my backpack ever since, now frayed after nearly ten years of being dragged on all sorts of experiences that my forever-eighteen-year-old friend will never have. Pink was his favorite color.

Pink was also my grandma’s favorite color. So much so that the couch that filled her living room for most of my childhood was unapologetically pink. Her bias toward the color became especially obvious between the months of November and February, when the couch stood beside an eight-foot-tall metallic pink Christmas tree, which she claimed doubled as a Valentine’s Day tree and was justified in staying up well into each new year. When she died during the spring of my senior year of college (probably only shortly after taking down the Christmas-tine’s tree), her funeral was filled with pink roses.

The question of what color felt like loss, therefore, seemed obvious. Especially because, until I started my clinical rotations, these two deaths were the only ones I had personally experienced. Pink is, for me, a natural reminder of those who I have lost and the efforts that I make to continue their memory.

Yet, when thinking of my deceased patients, I struggled with this. For two of my three patients, pink felt fitting. Perhaps not perfect, but not obviously wrong. But for the third patient, pink just wasn’t right. I only knew him briefly, but I felt like I knew him well enough to know that pink just wasn’t him. I talked to him and his family multiple times per day during his weeks-long hospitalization; we had conversations about what he wanted out of life and what gave his life meaning. His wife and I recounted the details of his cancer treatments again and again, searching for any possibility that he might qualify for a clinical trial. His teenage daughter and I shared a look after she shook her head and rolled her eyes when her dad claimed to have quit smoking “a while ago.” I knew the highs and lows of his five decades cut short and I knew what he wanted of his remaining days. And nothing I learned suggested that pink was a color that fit him whatsoever.

But maybe I was wrong. I only ever knew this patient from within the walls of the hospital. Perhaps, before the last weeks of his life, he was pink. Maybe when his daughter looked at him, she saw the pink cotton candy he got her at the ballpark when she was a kid instead of the ashen gray that had overtaken his skin. Maybe his wife’s memory of the pink flush that he brought to her cheeks when they danced was enough to overlook the deep red blood clots he passed as a complication of his late-stage cancer.

Even if they didn’t at that time, I hope they chose to remember him as pink. Because while pink, for me, is a color of loss, it is also the color of the living. This patient, and all the others, deserve to be remembered for their pink.

***

I got home shortly thereafter with a case of raspberry sparkling waters. I took one out of the case before pushing the rest to the back of the cabinet above the fridge – I didn’t want them to be mistakenly thrown in a lunchbox or offered as a refreshment while hosting friends. No, these pink cans were reserved for toasting the lives and deaths of my patients whose stories were finished. I fished out a fancy coupe glass I hadn’t used since starting medical school and poured what I could of the can into it.

Sitting at my kitchen counter, I made my first toast: to my three patients I cared for and ultimately lost while on Internal Medicine. It still didn’t feel quite right, but I realized that it probably wasn’t supposed to. Their deaths were devastating because their lives, as I learned through hours-long conversations with them and their families, were full of joy and love. I held the glass in front of me and observed the symbolism of its nature: more than half-full of liquid from a rose-colored can. I didn’t want this effort to color these losses as anything but exactly that: loss. Still, having been part of their final days, I knew that I would carry the memory of these patients with me for the rest of my career, and I hope only to honor their legacy in the care of my future patients. That started with taking a moment to remember them for their pink.

My toast wasn’t perfect, but it was a small gesture with a well-enjoyed but less-than-perfect flavor to honor the well-enjoyed lives and less-than-perfect deaths of these people who I knew too well and not at all.

